# The Effects of Mo and Nb on the Microstructures and Properties of CrFeCoNi(Nb,Mo) Alloys

**DOI:** 10.3390/e20090648

**Published:** 2018-08-29

**Authors:** Chun-Huei Tsau, Meng-Chi Tsai

**Affiliations:** Institute of Nanomaterials, Chinese Culture University, Taipei 111, Taiwan

**Keywords:** CrFeCoNi(Nb,Mo), microstructure, hardness, corrosion, sulfuric acid, sodium chloride

## Abstract

The effects of niobium and molybdenum additions on the microstructures, hardness and corrosion behaviors of CrFeCoNi(Nb,Mo) alloys were investigated. All of the CrFeCoNi(Nb,Mo) alloys displayed dendritic microstructures. The dendrites of CrFeCoNiNb and CrFeCoNiNb_0.5_Mo_0.5_ alloys were a hexagonal close packing (HCP) phase and the interdendrites were a eutectic structure of HCP and face-centered cubic (FCC) phases. Additionally, the dendrites of CrFeCoNiMo alloys were a simple cubic (SC) phase and the interdendrites were a eutectic structure of SC and FCC phases. The volume fraction of dendrites and interdendrites in these alloys were calculated. The influences of the volume fraction of dendrite in the alloys on the overall hardness were also discussed. The CrFeCoNiNb alloy had the larger volume fraction of dendrite and thus had the highest hardness among these alloys. The CrFeCoNi(Nb,Mo) alloys also showed better corrosion resistances in 1 M H_2_SO_4_ and 1 M NaCl solutions by comparing with commercial 304 stainless steel. The CrFeCoNiNb_0.5_Mo_0.5_ alloy possessed the best corrosion resistances in these solutions among the CrFeCoNi(Nb,Mo) alloys.

## 1. Introduction

High-entropy alloys (HEA) has been announced for more than ten years [1,2,3]. The concept of HEA provides a new field for alloys design and thus becomes a very important field of materials development. This high-entropy alloy concept is now widely used to develop the high-performance alloys [4,5] and refractory alloys [6,7], also it is also applied in the thin film processes [8,9,10]. All of these researches are focus on the unique properties of the high-entropy alloys. Corrosion resistance is an important property of high-entropy alloys for structural applications; and many high-entropy alloys possess good corrosion resistances in different solutions are reported, such as FeCoNiCrCu*_x_* high-entropy alloys in 3.5% sodium chloride solution [11], Al_7.5_Cr_22.5_Fe_35_Mn_20_Ni_15_ high-entropy alloy in different solutions [12] and Al_0.5_CoCrFeNi alloy in a 3.5% NaCl solution [13]. That is, the high-entropy alloy concept is used to develop structural alloys with good corrosion resistance.

CrFeCoNi alloy has a very good corrosion resistance property is reported in our previous study [14]. It has a granular FCC structure and some HCP precipitates. However, the hardness of CrFeCoNi alloy is too low (HV144) to limit its structural application. Molybdenum has a benefit on the corrosion resistance of stainless steels is well known [15,16]. Niobium also can improve the corrosion resistance of FeCuNbSiB amorphous alloys [17]. Therefore, this study adds Mo and/or Nb into CrFeCoNi alloy and tests their properties. The microstructures revolution, hardness and polarization behaviors of corrosion of the CrFeCoNi(Nb,Mo) alloys in H_2_SO_4_ and NaCl solutions are all tested to evaluate their commercial application.

## 2. Experimental

The CrFeCoNiNb, CrFeCoNiNb_0.5_Mo_0.5_ and CrFeCoNiMo alloys were prepared by arc melting using appropriate amounts of the elements with purities above 99.9%. The alloys were made under a partial pressure of argon atmosphere (400 torrs). The bottoms were remelted at least 4 times to ensure homogeneity. Table 1 lists the chemical compositions of the alloys, the maximum deviation of each element in the alloys was less than 1 atomic percent. The microstructural evolution of the as-cast alloys was observed using a field emission scanning electron microscope with an energy dispersive spectrometer (SEM/EDS, JEOL JSM-6335, JEOL Ltd., Tokyo, Japan), which was operated at 15 kV. The structures were characterized by X-ray diffraction (XRD) using a Rigaku ME510-FM2 (Rigaku Ltd., Tokyo, Japan) with Cu-K (with a wavelength of 1.5406 Å) radiation operated at 30 kV at a scanning rate of 0.04 degree/s. The microstructures and lattice images of the alloys were obtained using a high-resolution transmission electron microscope (HREM, JEOL JEM-3000F, JEOL Ltd., Tokyo, Japan), which was operated at 300 kV. The corresponding diffraction patterns (DP) were obtained from the high-resolution lattice images by fast Fourier transformation (FFT) in Gatan digital micrograph software. The hardness of the alloys was measured using both a Mitutoyo Akashi MVK-G1500 microhardness tester (Mitutoyo Co., Kanagawa, Japan) under a load of 10 gf and a Matsuzawa Seiki MV1 Vicker’s hardness tester (Matsuzawa Co., Akita, Japan) under a load of 30 kgf.

Polarization curves of the as-cast alloys were obtained in a potentiostat/galvanostat (Autolab PGSTAT302N, Metrohm Autolab B.V., Utrecht, The Netherlands) using a three-electrode system at a scanning rate of 1 mV/s. The CrFeCoNi(Nb,Mo) alloys for polarization testing were mounted in epoxy resin and the exposed surface area of each was fixed at 19.64 mm^2^ (with a diameter of 5 mm). The reference electrode was a saturated silver chloride electrode (Ag/AgCl) and the counter electrode was a smooth Pt sheet. All the potentials that are below a saturated silver chloride electrode (SSE), whose reduction potential is 222 mV higher than that of the standard hydrogen electrode (SHE) at 25 °C [18]. The specimens whose polarization curves were obtained were all mechanically wet-polished using 1200 SiC grit paper. Test solutions with a concentration of 1 M were prepared from reagent-grade sulfuric acid (H_2_SO_4_) and sodium chloride (NaCl) that were dissolved in distilled water. To eliminate any effect of dissolved oxygen, the solutions were deaerated by bubbling nitrogen gas through them before and during the polarization experiments. The polarization test started after the specimen, counter electrode and reference electrode were placed in the bubbling solution for 900 s.

## 3. Results and Discussion

**Figure 1:**
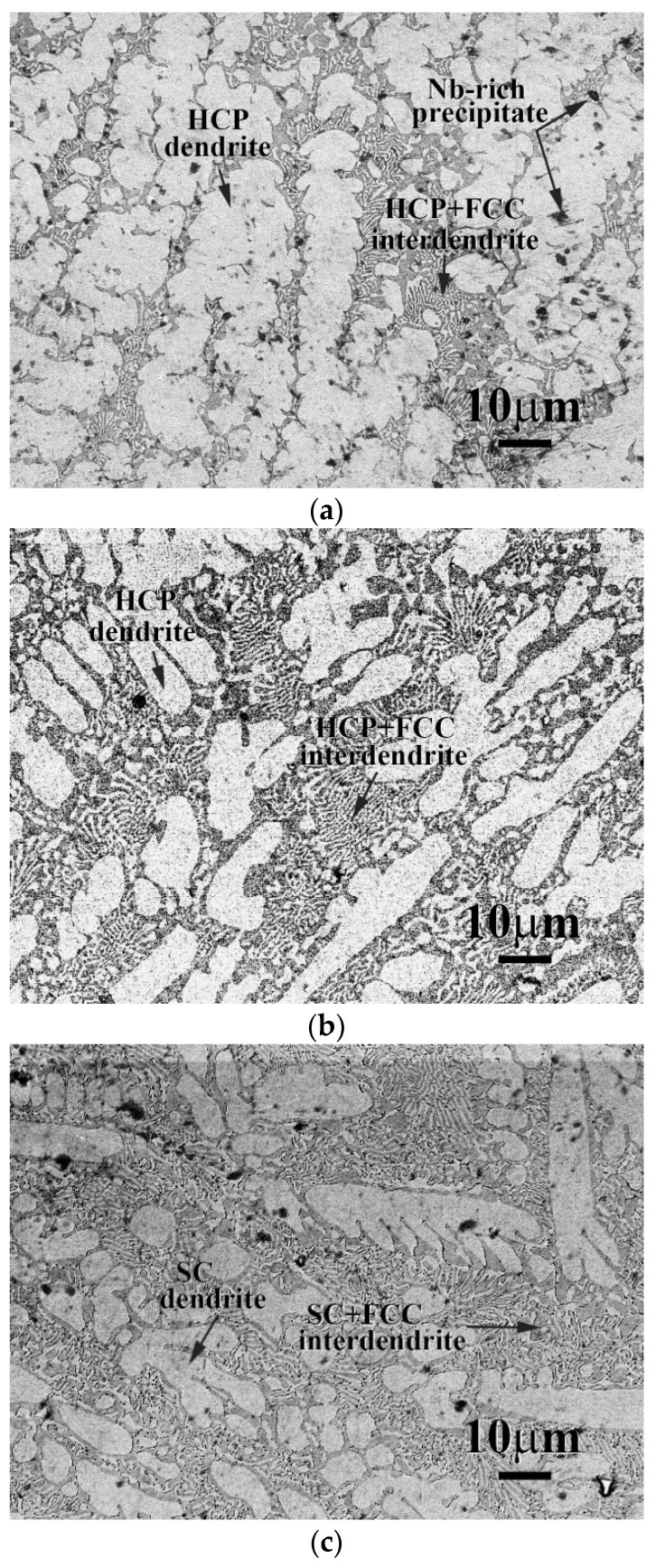
The SEM micrographs of as-cast (**a**) CrFeCoNiNb; (**b**) CrFeCoNiNb_0.5_Mo_0.5_; and (**c**) CrFeCoNiMo alloys.

The microstructures of as-cast CrFeCoNi(Nb,Mo) alloys are displayed in Figure 1. They indicated that the Nb and Mo additions could change the granular microstructures of CrFeCoNi alloy to a dendritic microstructure of CeFeCoNi(Nb,Mo) alloys; and their interdendrites all showed a eutectic structure. Also, some precipitates were only observed in the as-cast CrFeCoNiNb alloy, shown in Figure 1a; the precipitates were not found in the other two alloys under the as-cast state. The chemical compositions of the phases in these CrFeCoNi(Nb,Mo) alloys are listed in Table 2. The deviation of each element-content in the phases were less than 1 atomic percent. The dendrites of these three alloys had higher niobium and/or molybdenum contents. Additionally, the precipitates in CrFeCoNiNb alloy had similar compositions with the HCP dendrites but the lattice constants of these two phases were quite different.

**Figure 2:**
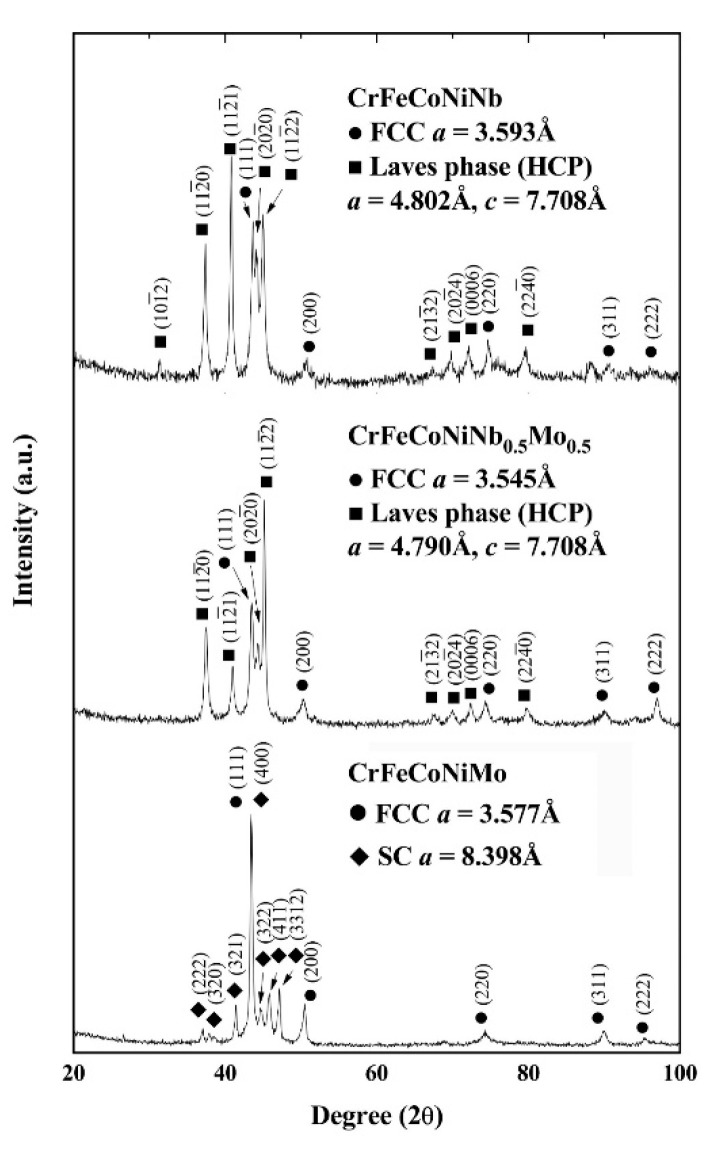
X-ray diffraction (XRD) patterns of the as-cast CrFeCoNi(Nb,Mo) alloys.

Figure 2 shows the XRD patterns of the as-cast CrFeCoNi(Nb,Mo) alloys. The lattice constants of the phases are also marked in the figure. It indicated that every alloy had two major phases. The major phases in the as-cast CrFeCoNiNb and CrFeCoNiNb_0.5_Mo_0.5_ alloys were the FCC and HCP phases, the lattice constants of both the FCC and HCP phases in these two alloys were also very close. No peak of the precipitates was found in the as-cast CrFeCoNiNb alloy because its volume fraction was too small. However, the phases in the as-cast CrFeCoNiMo alloy were an FCC phase and a simple cubic (SC) phase, the SC phase (isometric cubic) had a large lattice constant of 8.398 Å which indicated that a unit cell had many atoms.

**Figure 3:**
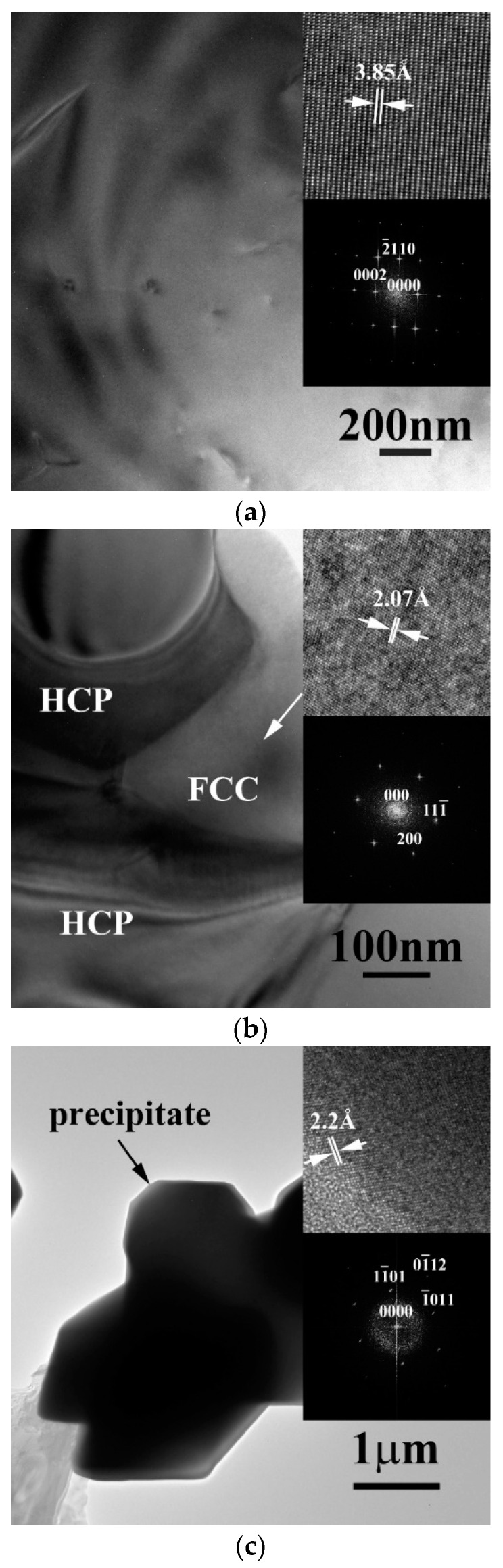
Transmission electron microscopy (TEM) (bright field) BF images of the phases in as-cast CrFeCoNiNb alloy: (**a**) an image of dendrite, inserts are the corresponding lattice image and fast Fourier transformation (FFT) diffraction patterns (DP) taken from the zone axis of [01$\bar{1}$0] which shows a hexagonal close packing (HCP) structure; (**b**) an image of interdendrite, inserts are the corresponding lattice image and FFT DP taken from the zone axis of [011] which shows an FCC structure; and (**c**) an image of precipitate, inserts are the corresponding lattice image and FFT DP taken from the zone axis of [01$\bar{1}$1] which shows a HCP structure.

The transmission electron microscopy (TEM) bright field (BF) images of the dendrite, the matrix of the interdendrite and the precipitate of as-cast CrFeCoNiNb alloy are shown in Figure 3a–c, respectively. Figure 3a displays the image of the dendrite and inserts are the corresponding lattice image and the FFT DP, which were taken from the zone axis of [01$\bar{1}$0]; and the FFT DP indicates that the dendrite was a single HCP phase. Figure 3b is the TEM image of the matrix of interdendrite in the alloy, the inserts are the corresponding lattice image and FFT DP, which were taken from the zone zxis of [011]; and this FFT DP indicated that it was an FCC structure. Figure 3c shows the TEM image of the precipitate and its corresponding lattice image and FFT DP, which were taken from the zone axis of [01$\bar{1}$1]. The precipitates showed a HCP structure and its lattice constants of *a*- and *c*-axes were 2.88 and 4.70 Å, respectively.

**Figure 4:**
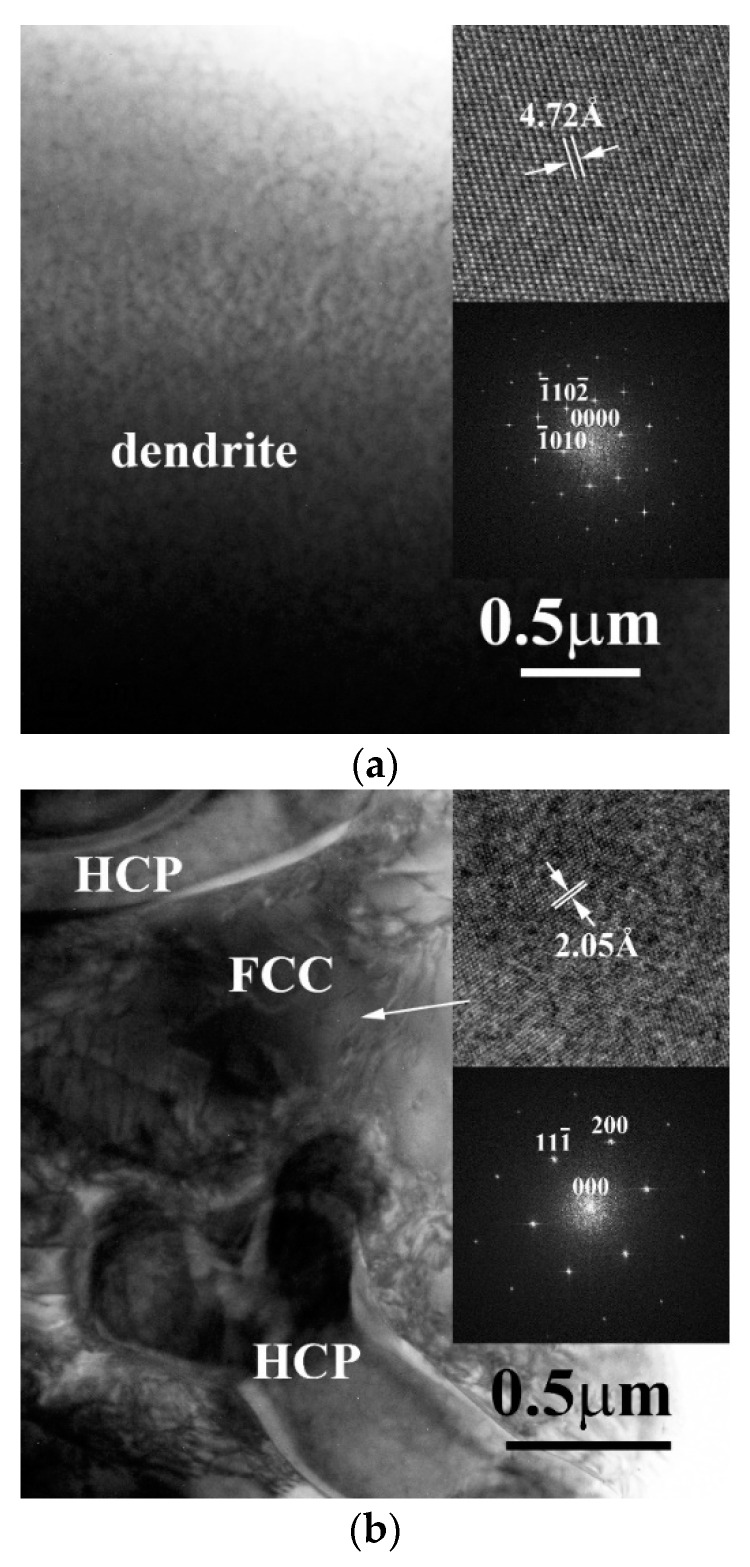
TEM BF images of the phases in as-cast CrFeCoNiNb_0.5_Mo_0.5_ alloy: (**a**) an image of dendrite, inserts are the corresponding lattice image and FFT DP taken from the zone axis of [$\bar{2}$4$\bar{2}$3] which shows a HCP structure; (**b**) an image of interdendrite, inserts are the corresponding lattice image and FFT DP taken from the zone axis of [011] which shows an FCC structure.

Figure 4a shows the TEM BF images of the dendrite in CrFeCoNiNb_0.5_Mo_0.5_ alloy, inserts are the corresponding lattice image and FFT DP, which were taken from the zone axis of [$\bar{2}$4$\bar{2}$3]; and this FFT DP indicated that this phase was a HCP structure. Figure 4b show the TEM BF image of the interdendrite in CrFeCoNiNb_0.5_Mo_0.5_ alloy, inserts are the corresponding lattice image and FFT DP, which were taken from the zone axis of [011]; and this FFT DP indicated an FCC structure. 

**Figure 5:**
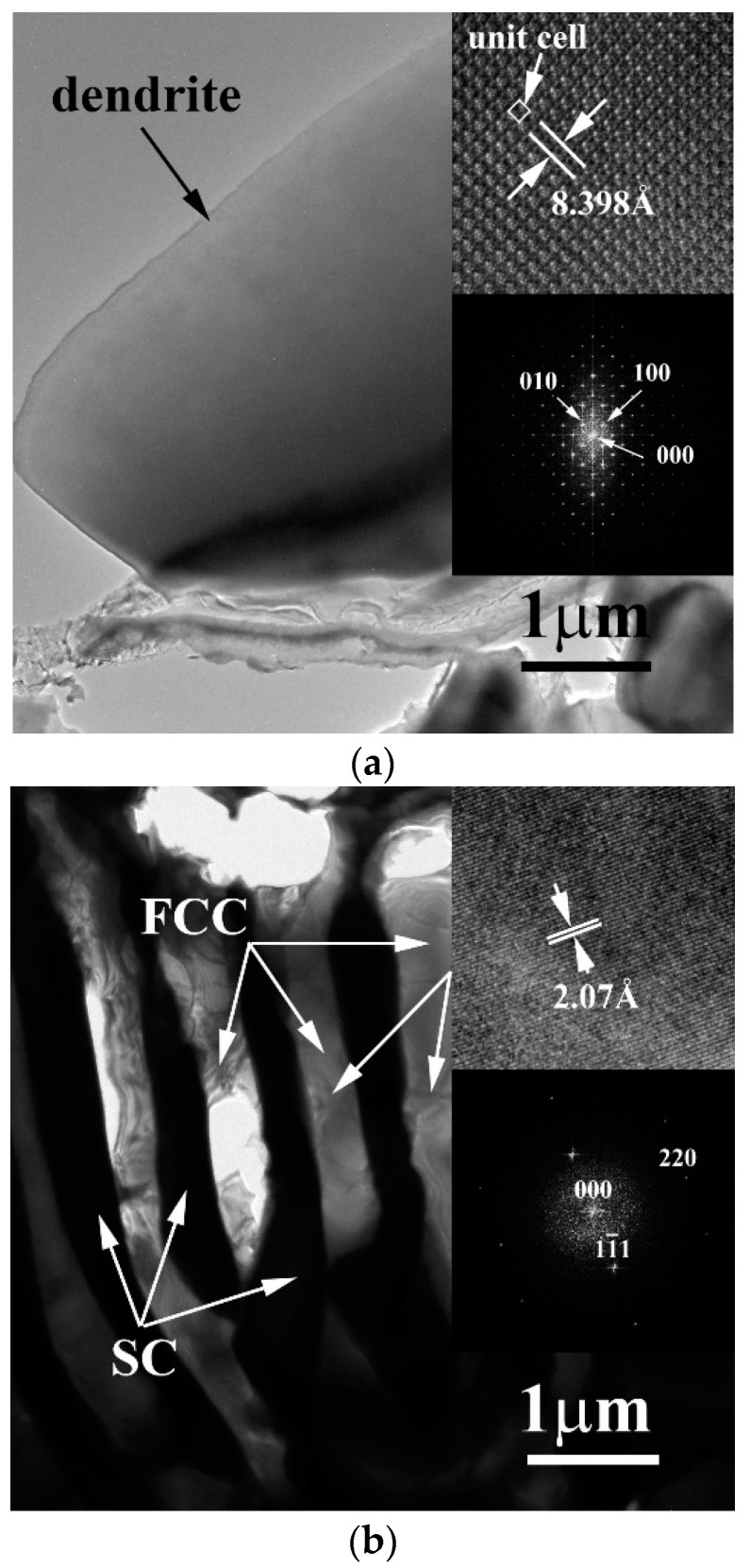
TEM BF images of the phases in as-cast CrFeCoNiMo alloy: (**a**) an image of dendrite, inserts are the corresponding lattice image and FFT DP taken from the zone axis of [001] which shows a SC structure; (**b**) an image of interdendrite, inserts are the corresponding lattice image and FFT DP taken from the zone axis of [$\bar{1}$12] which shows an FCC structure.

Figure 5a shows a TEM BF image of the dendrite of as-cast CrFeCoNiMo alloy, inserts are the corresponding lattice image and FFT DP, which were taken from the zone axis of [001]. The image indicated that the dendrite was a single phase. However, the lattice image indicated that the unit cell of this phase had a large lattice constant of 8.398 Å. Therefore, the diffraction spots of the FFT DP were very close which indicated that this phase had a large lattice constant. Additionally, the lattice points from the lattice image showed only 1-fold symmetry and thus the unit cell had a SC structure (i.e., an isometric cubic structure) with a large lattice constant. This also meant the SC structure had a complex structure, this complex structure needs further investigation. Figure 5b displays the image of the eutectic interdendrite in the as-cast CrFeCoNiMo alloy. Both of the corresponding lattice image and FFT DP taken from the zone axis of [$\bar{1}$12] confirmed that the matrix of interdendrite was an FCC structure.

The influence of niobium and molybdenum additions on the volume fractions of the dendrites and interdendrites of these alloys were different. Table 3 lists the volume fraction of the dendrites of as-cast CrFeCoNi(Nb,Mo) alloys. To determine of the volume fraction of dendrites was by drawing arbitrary lines in photos and measuring the intercept lengths of intercepted dendrites. From this, the volume fraction of the dendrites was calculated by the equation [19]:

(1) $$V_{d}=L_{d}=\frac{\sum L_{a}}{L_{T}}$$

where *V_d_* is the volume fraction of the dendrites, *L_d_* is the linear fraction of the dendrites, *L_a_* is the intercepted length of each dendrite and *L_T_* is the total length. The results indicated that the CrFeCoNiNb alloy had more volume fraction of dendrites; and molybdenum addition would decrease the volume fraction of dendrites.

The overall hardness of as-cast CrFeCoNi(Nb,Mo) alloys and the microhardness of the dendrites and interdendrites of the alloys are list in Table 4. The hardness of as-cast CrFeCoNi alloy only had HV 144. However, the hardness increased sharply after additions of niobium and/or molybdenum. Because of niobium and molybdenum had larger atomic radiuses. The atomic radiuses of Cr, Fe, Co, Ni, Nb and Mo are 0.128, 0.124, 0.125, 0.125, 0.143 and 0.140 nm, respectively [20]. Therefore, the hardness increased significantly because of the larger lattice distortion and forming the different phases after additions of niobium and molybdenum. The interdendrites of the alloys had almost the same hardness (about HV 400). The dendrites of the alloys were harder than the interdendrites (about HV 700). The overall hardness of an alloy was contributed by the volume fractions of the dendrite (the hard part) and the interdendrite (the soft part) in this alloy. Therefore, decreasing the volume fraction of the hard part, that is, the dendrites, would result in decreasing the overall hardness of the alloy. Both the dendrites and interdendrites of CrFeCoNiNb_0.5_Mo_0.5_ alloy had the highest hardness but the overall hardness of this alloy was lowest among these alloys. This was contributed by the lowest volume fraction of dendrite (the hard part) in the CrFeCoNiNb_0.5_Mo_0.5_ alloy. On the contrary, the CrFeCoNiNb alloy had the highest volume fraction of dendrite and thus had the highest overall hardness among these alloys.

**Figure 6:**
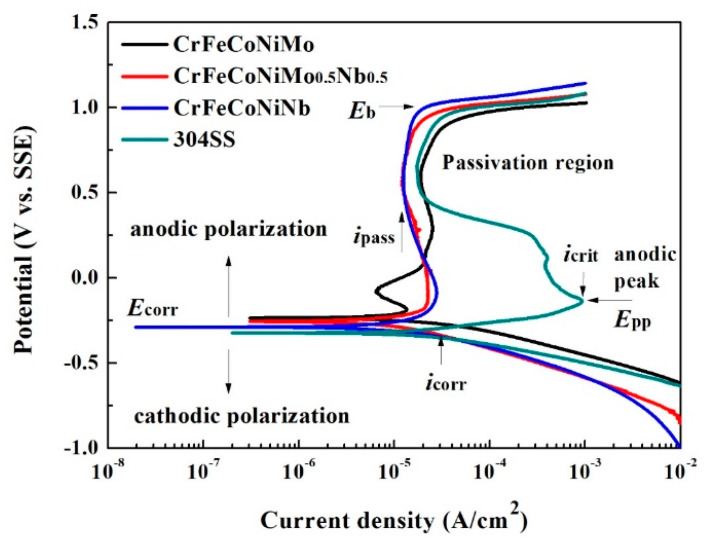
Polarization curves of CrFeCoNi(Nb,Mo) alloys and 304 stainless steel in 1 M deaerated H_2_SO_4_ solution at 30 °C.

The polarization behaviors of the as-cast CrFeCoNi(Nb,Mo) alloys and 304 stainless steel in 1M deaerated H_2_SO_4_ solution at 30 °C are shown in Figure 6. The polarization data were also compared with those of commercial 304 stainless steel (304SS) whose composition was by weight 71.61% Fe, 18.11% Cr, 8.24% Ni, 1.12% Mn, 0.75% Si, 0.05% Co, 0.02% Mo, 0.05% C, 0.03% P and 0.02% S. The important data of these polarization curves are listed in Table 5. The corrosion potential (*E*_corr_) of CrFeCoNi(Nb,Mo) alloys were very close and all nobler than that of 304SS. Table 6 lists the standard electrode potential of selected elements [21]. The standard electrode potential of niobium is lower than that of molybdenum, which means that the niobium is more active than molybdenum. Therefore, the CrFeCoNiMo alloy had the highest *E*_corr_ and the CrFeCoNiNb alloy had the lowest *E*_corr_ among these three alloys. The polarization curve below *E*_corr_ was the cathodic polarization curve; and the curve above *E*_corr_ was the anodic polarization curve. The corrosion current densities (*i*_corr_) of CrFeCoNi(Nb,Mo) alloys were less or equal to the *i*_corr_ of 304SS. The polarization curve of 304 stainless steel displayed a large anodic peak; and the anodic peaks of CrFeCoNi(Nb,Mo) alloys were significantly less than that of 304SS and thus CrFeCoNi(Nb,Mo) alloys had lower passivation potential (*E*_pp_) and anodic critical current density (*i*_crit_). This meant that the CrFeCoNi(Nb,Mo) alloys were easy to enter passivation regions and form passive films during corrosion in H_2_SO_4_ solution by comparing with 304 stainless steel. The large anodic peak of 304 stainless steel in H_2_SO_4_ solution was caused by formation of iron hydroxide and higher oxides of iron and chromium [22]. The lowest current densities of the passivation regions (*i*_pass_) of these alloys were around 10–20 A/cm^2^. Additionally, the main passivation region of CrFeCoNi(Nb,Mo) alloys were broader than that of 304SS. All of these alloys had a similar breakdown potential (*E*_b_) of about 1 V (SSE).

**Figure 7:**
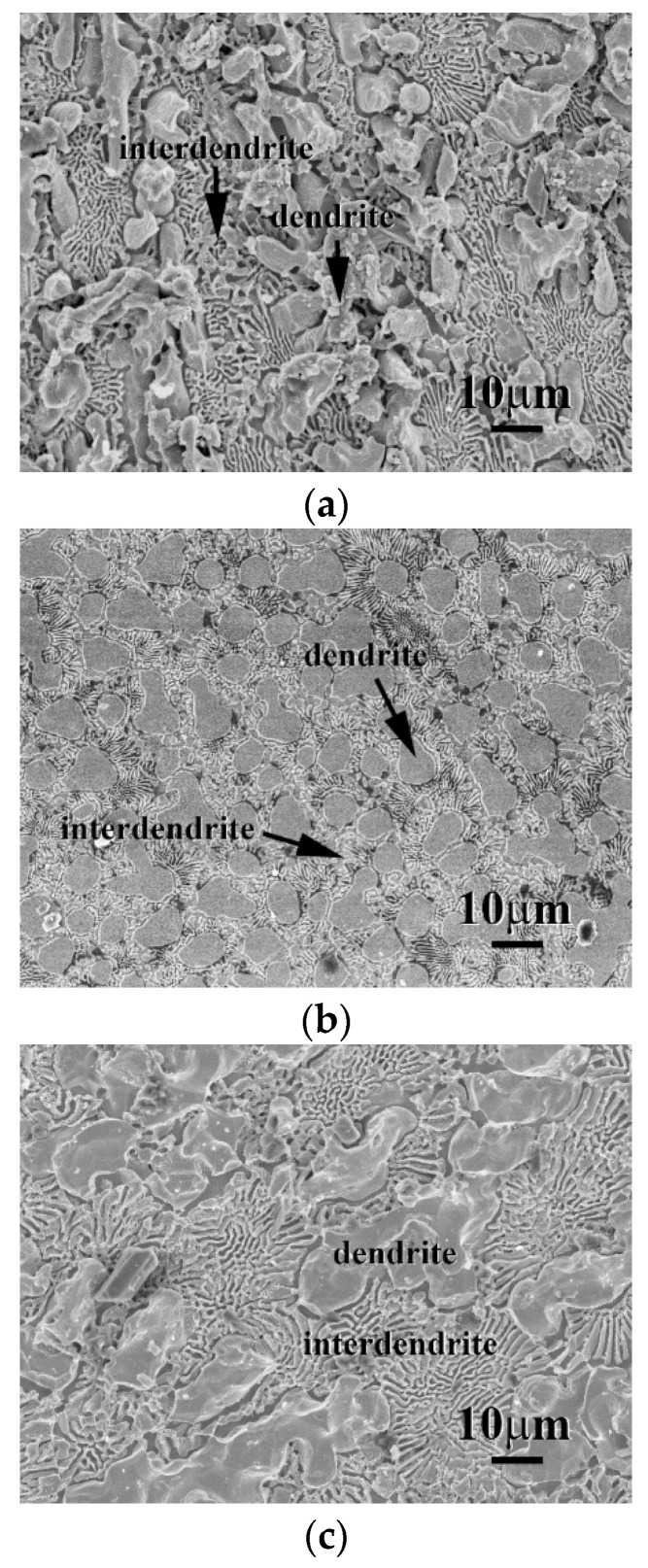
SEM micrographs of the alloys after polarization test in 1 M deaerated H_2_SO_4_ solution at 30 °C, (**a**) CrFeCoNiNb alloy; (**b**) CrFeCoNiNb_0.5_Mo_0.5_ alloy; and (**c**) CrFeCoNiMo alloy.

The micrographs of CrFeCoNi(Nb,Mo) alloys after the polarization test in 1 M deaerated H_2_SO_4_ solution at 30 °C are shown in Figure 7. Both of the dendrites and interdendrites of CrFeCoNiNb alloy were significantly corroded after test, as shown in Figure 7a; but the FCC phase (the matrix of interdendrite) was severely corroded than the HCP phase. On the contrary, only the FCC phase (the matrix of interdendrite) of CrFeCoNiNb_0.5_Mo_0.5_ alloy was slightly corroded, the HCP phase almost maintained its original shape, as shown in Figure 7b. This also proved that the CrFeCoNiNb_0.5_Mo_0.5_ alloy had the minimum *i*_corr_ among these CrFeCoNi(Nb,Mo) alloys, as listed in Table 5. The micrograph of CrFeCoNiMo alloy also displayed a severely corroded surface after polarization test, as shown in Figure 7c. Also, the FCC phase (the matrix of interdendrite) was more corroded than the SC phase. Therefore, in the local cells of the CrFeCoNi(Nb,Mo) alloys, the FCC phase in the interdendrites behaved as an anode and another phase (e.g., the HCP phase of CrFeCoNiNb and CrFeCoNiNb_0.5_Mo_0.5_ alloys and the SC phase of CrFeCoNiMo alloy) behaved as a cathode.

**Figure 8:**
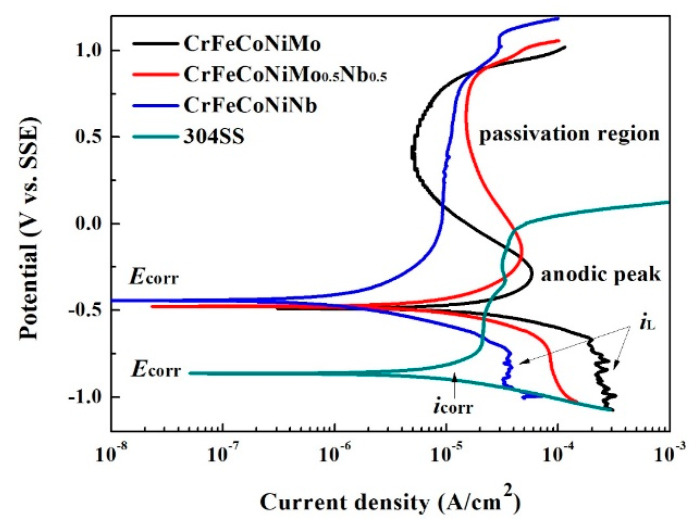
Polarization curves of CrFeCoNi(Nb,Mo) alloys and 304 stainless steel in 1 M deaerated NaCl solution at 30 °C.

The polarization curves of the as-cast CrFeCoNi(Nb,Mo) alloys in 1 M deaerated NaCl solution at 30 °C are shown in Figure 8. The values of *E*_corr_ and *i*_corr_ of these alloys are listed in Table 7. All of these data are also compared with commercial 304 stainless steel. The *i*_corr_ of these four alloys were also close. In addition, the *E*_corr_ of the CrFeCoNi(Nb,Mo) alloys were very close and much nobler than 304 stainless steel. However, the passivation regions of CrFeCoNi(Nb,Mo) alloys were much broader than that of 304 stainless steel. Both of the CrFeCoNiMo and CrFeCoNiNb_0.5_Mo_0.5_ alloys had significantly anodic peaks and passivation regions; but CrFeCoNiNb and 304 stainless steel did not display anodic peaks. Adding Mo can reportedly increase the corrosion resistance of the alloy in a solution that contains chloride ions because molybdenum can increase the stability of the passivation films of steels [15,23]. The polarization curves of CrFeCoNi(Nb,Mo) alloys indicated that the increasing of Mo-content resulted in forming anodic peak and passivation regions. In addition, the cathodic limiting current densities (*i*_L_) were observed in the polarization curves of CrFeCoNiNb and CrFeCoNiMo alloys. The cathodic limiting current density (*i*_L_) related to the maximum reaction rate, which was limited by the diffusion rate of hydroxyl ions (OH^−^) in solution [18].

**Figure 9:**
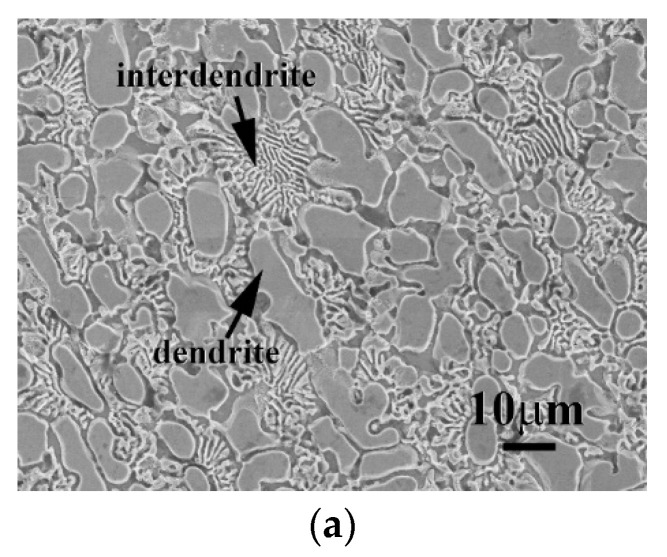

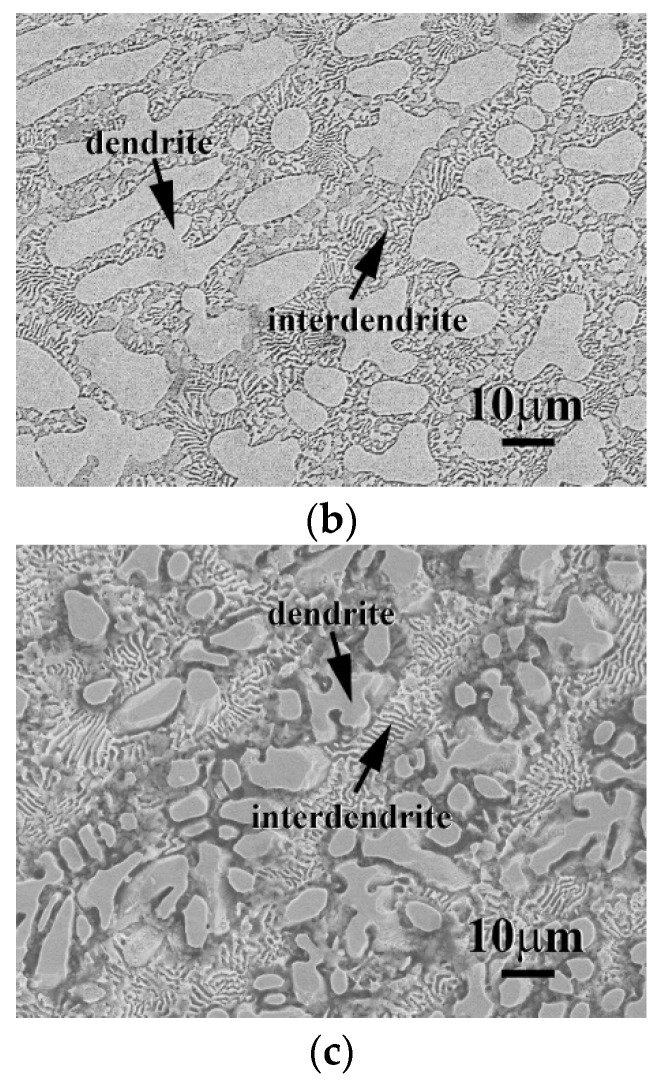
SEM micrographs of the alloys after polarization test in 1 M deaerated NaCl solution at 30 °C, (**a**) CrFeCoNiNb alloy; (**b**) CrFeCoNiNb_0.5_Mo_0.5_ alloy; and (**c**) CrFeCoNiMo alloy.

The micrographs of the as-cast CrFeCoNi(Nb,Mo) alloys after polarization test in 1 M deaerated NaCl solution at 30 °C are shown in Figure 9. Similar to the results of these alloys tested in 1 M deaerated H_2_SO_4_ solution at 30 °C, the major corroded areas of these alloys were the matrixes of the interdendrites (FCC phase) of CrFeCoNi(Nb,Mo) alloys. On the contrary, almost no corrosion occurred on the dendrites of these CrFeCoNi(Nb,Mo) alloys. Furthermore, no deep-type pitting was observed indicated that these alloys had a good corrosion resistance in NaCl solution. This also proved that molybdenum could improve the localized corrosion resistance.

## 4. Conclusions

All of the CrFeCoNi(Nb,Mo) alloys displayed dendritic microstructures. The major two phases of CrFeCoNiNb and CrFeCoNiNb_0.5_Mo_0.5_ alloys were the HCP and FCC phases, where the dendrites were a single HCP phase. The major two phases of CrFeCoNiMo alloys were the SC and FCC phases, where the dendrites were a single SC phase. All of the interdendrites in the CrFeCoNi(Nb,Mo) alloys were eutectic structures.

The microhardness and overall hardness of CrFeCoNi(Nb,Mo) alloys increased by comparing with CrFeCoNi alloy because the elements of niobium and molybdenum had larger atomic radiuses. The microstructures significantly influenced the overall hardness of these alloys. The highest overall hardness of CrFeCoNiNb alloy was caused by its larger volume fraction of the dendrites. On the contrary, the lowest overall hardness of CrFeCoNiNb_0.5_Mo_0.5_ alloy was caused by its less volume fraction of the dendrites.

The corrosion resistances of CrFeCoNi(Nb,Mo) alloys in 1M deaerated H_2_SO_4_ and NaCl solutions were better than commercial 304 stainless steel. Additionally, the CrFeCoNiNb_0.5_Mo_0.5_ alloy had the best corrosion resistances in these solutions form the polarization curves and the micrographs after corrosion test. In these CrFeCoNi(Nb,Mo) alloys, the FCC phase behaved as an anode of the local cell in the alloy and was thus severely corroded than another phase in 1M deaerated H_2_SO_4_ and NaCl solutions.

## Figures and Tables

**Table 1:** The average chemical compositions of the as-cast CrFeCoNi(Nb,Mo) alloys analyzed by SEM/EDS.

Alloys	Compositions (Atomic Percent)					
	Cr	Fe	Co	Ni	Nb	Mo
CrFeCoNiMo	20.1	20.2	19.5	20.5	N/A	19.7
CrFeCoNiNb_0.5_Mo_0.5_	21.4	19.4	19.8	17.6	11.0	10.8
CrFeCoNiNb	19.8	19.8	19.0	19.5	22.0	N/A

**Table 2:** The average chemical compositions of the phases in the CrFeCoNi(Nb,Mo) alloys analyzed by SEM/EDS.

Alloys	Compositions (Atomic Percent)					
	Co	Cr	Fe	Ni	Mo	Nb
CrFeCoNiNb						
FCC	18.4	26.7	24.3	25.5	N/A	5.1
HCP	19.7	17.1	18.1	14.8	N/A	30.3
precipitate	19.1	16.7	16.7	15.7	N/A	31.8
CrFeCoNiNb_0.5_Mo_0.5_						
FCC	18.4	22.1	20.4	22.8	9.2	7.1
HCP	19.1	17.3	23.6	14.2	15.6	16.9
CrFeCoNiMo						
FCC	21.3	19.2	22.0	24.0	13.5	N/A
SC	17.2	21.9	17.7	14.4	28.9	N/A

**Table 3:** The volume fraction of the dendrites in the as-cast CrFeCoNi(Nb,Mo) alloys.

Alloys	Volume Fraction of the Dendrites (vol.%)
CrFeCoNiMo	42 ± 6
CrFeCoNiNb_0.5_Mo_0.5_	36 ± 6
CrFeCoNiNb	68 ± 4

**Table 4:** The hardness of the CrFeCoNi(Nb,Mo) alloys.

Alloys	Hardness		
	Overall	Dendrite	Interdendrite
CrFeCoNiMo	604 ± 8	692 ± 18	405 ± 9
CrFeCoNiNb_0.5_Mo_0.5_	533 ± 6	745 ± 10	412 ± 7
CrFeCoNiNb	652 ± 8	693 ± 23	398 ± 24

**Table 5:** Polarization data of CrFeCoNi(Nb,Mo) alloys and 304 stainless steel in 1 M deaerated H_2_SO_4_ solution at 30 °C.

Alloys	*i*_corr_ μA/cm^2^	*E*_corr_ V vs. SSE	*E*_pp_ V vs. SSE	*i*_crit_ mA/cm^2^	*i_pass_* μA/cm^2^
CrFeCoNiNb	22.3	−0.290	−0.090	0.028	12.4
CrFeCoNiNb_0.5_Mo_0.5_	12.9	−0.256	−0.165	0.022	12.2
CrFeCoNiMo	30.0	−0.236	−0.174	0.013	18.9
304SS	30.0	−0.320	−0.140	0.930	17.2

**Table 6:** Standard electrode potential at 25 °C [21].

Reaction	Electrode Potential (*E*° vs. SSE)
Cr, Cr^3+^	−0.962
Fe, Fe^2+^	−0.662
Co, Co^2+^	−0.449
Ni, Ni^2+^	−0.472
Nb, Nb^3+^	−1.322
Mo, Mo^3+^	−0.422

**Table 7:** Polarization data (*i*_corr_ and *E*_corr_) of CrFeCoNi(Nb,Mo) alloys and 304 stainless steel in 1 M deaerated NaCl solution at 30 °C.

Alloys	*i*_corr_	*E*_corr_
	A/cm^2^	V vs. SSE
CrFeCoNiNb	1.2	−0.443
CrFeCoNiNb_0.5_Mo_0.5_	6.7	−0.477
CrFeCoNiMo	13.0	−0.489
304SS	12.9	−0.860

## References

[1] Yeh J.W., Chen S.K., Lin S.J., Gan J.Y., Chin T.S., Shun T.T., Tsau C.H., Chang S.Y. (2004). Nanostructured high-entropy alloys with multiple principal elements: Novel alloy design concepts and outcomes. Adv. Eng. Mater..

[2] Murty B.S., Yeh J.W., Ranganathan S. (2014). High-Entropy Alloys.

[3] Zhang W., Liaw P.K., Zhang Y. (2018). Science and technology in high-entropy alloys. Sci. China Mater..

[4] Wu J.M., Lin S.J., Yeh J.W., Chen S.K., Huang Y.S., Chen H.C. (2006). Adhesive wear behavior of Al_x_CoCrCuFeNi high-entropy alloys as a function of aluminum content. Wear.

[5] Huo W.Y., Shi H.F., Ren X., Zhang J.Y. (2015). Microstructure and wear behavior of CoCrFeMnNbNi High-Entropy Alloy Coating by TIG Cladding. Adv. Mater. Sci. Eng..

[6] Senkov O.N., Wilks G.B., Scott J.M., Miracle D.B. (2011). Mechanical properties of Nb_25_Mo_25_Ta_25_W_25_ and V_20_Nb_20_Mo_20_Ta_20_W_20_ refractory high entropy alloys. Intermetallics.

[7] Gao M.C., Carney C.S., Doğan Ő.N., Jablonksi P.D., Hawk J.A., Alman D.E. (2015). Design of refractory high-entropy alloys. JOM.

[8] Antonaglia J., Xie X., Tang Z., Tsai C.W., Qiao J.W., Zhang Y., Laktionova M.O., Tabachnikova E.D., Yeh J.W., Senkov O.N. (2014). Temperature effects on deformation and serration behavior of high-entropy alloys (HEAs). JOM.

[9] Sheng W.J., Yang X., Wang C., Zhang Y. (2016). Nano-Crystallization of high-entropy amorphous NbTiAlSiW_x_N_y_ films prepared by magnetron sputtering. Entropy.

[10] Zhang Y., Yan X.-H., Liao W.-B., Zhao K. (2018). Effects of nitrogen content on the structure and mechanical properties of (Al_0.5_CrFeNiTi_0.25_)Nx high-entropy films by reactive sputtering. Entropy.

[11] Hsu Y.J., Chiang W.C., Wu J.K. (2005). Corrosion behavior of FeCoNiCrCu*_x_* high-entropy alloys in 3.5% sodium chloride solution. Mater. Chem. Phys..

[12] Tsau C.H., Lee P.Y. (2016). Microstructures of Al_7.5_Cr_22.5_Fe_35_Mn_20_Ni_15_ high-entropy alloy and its polarization behaviors in sulfuric acid, nitric acid and hydrochloric acid solutions. Entropy.

[13] Lin C.M., Tsai H.L. (2011). Evolution of microstructure, hardness, and corrosion properties of high-entropy Al_0.5_CoCrFeNi alloy. Intermetallics.

[14] Tsau C.H., Lin S.X., Fang C.H. (2017). Microstructures and corrosion behaviors of FeCoNi and CrFeCoNi equimolar alloys. Mater. Chem. Phys..

[15] Hashimoto K., Asami K., Teramoto K. (1979). An X-ray photo-electron spectroscopic study on the role of molybdenum in increasing the corrosion resistance of ferritic stainless steels in HCl. Corros. Sci..

[16] Pardo A., Merino M.C., Coy A.E., Viejo F., Arrabal R., Matykina E. (2008). Effect of Mo and Mn additions on the corrosion behavior of AISI 304 and 316 stainless steel in H_2_SO_4_. Cossos. Sci..

[17] Mariano N.A., Souza C.A.C., May J.E., Kuri S.E. (2003). Influence of Nb content on the corrosion resistance and saturation magnetic density of FeCuNbSiB alloys. Mater. Sci. Eng. A.

[18] Revie R.W., Uhlig H.H. (2008). Corrosion and Corrosion Control: An Introduction to Corrosion Science and Engineering.

[19] Voort G.F.V. (1999). Metallography-Principles and Practice.

[20] Smith W.F. (2004). Foundations of Materials Science and Engineering, 3rd ed.

[21] Chawla S.L. (1993). Materials Selection for Corrosion Control.

[22] Abdallah M. (2003). Corrosion behavior of 304 stainless steel in sulphuric acid solutions and its inhibition by some substituted pyrazolones. Mater. Chem. Phys..

[23] Tomio A., Sagara M., Doi T., Amaya H., Otsuka N., Kudo T. (2015). Role of alloyed molybdenum on corrosion resistance of austenitic Ni-Cr-Mo-Fe alloys in H_2_S-Cl^−^ environments. Corros. Sci..

